# Association between polymorphism near the *MC4R* gene and cancer risk: A meta-analysis

**DOI:** 10.1097/MD.0000000000022003

**Published:** 2020-09-04

**Authors:** Tian Zeng, Jing Zhao, Yu Kang, Xiaojiao Wang, Hongjun Xie

**Affiliations:** a Medical College, Tibet University, Lhasa, China; b Department of Oncology, Southwest Hospital, Army Medical University, Chongqing, China.

**Keywords:** cancer, *MC4R*, meta-analysis, single nucleotide polymorphism

## Abstract

**Objective:**

Genome-wide association studies have identified single nucleotide polymorphisms (SNPs) near the *melanocortin 4 receptor* (*MC4R*), gene which are associated with risk of obesity. Since obesity is an established risk factor of cancer, several studies have examined the association between SNPs near the *MC4R* gene and cancer risk, but the findings are inconsistent. The present study aimed to perform a meta-analysis to clarify the association between SNPs near *MC4R* and cancer risk.

**Methods:**

The PubMed and Embase databases were searched for potentially eligible publications. All studies that evaluated the association between *MC4R* rs17782313 SNP (or its proxy rs12970134) and cancer risk were included. The pooled odds ratios with 95% confidence intervals (CIs) were calculated using the random-effects model. And subgroup analysis by cancer type (colorectal cancer, endometrial cancer and breast cancer) was conducted for further investigate the association.

**Results:**

A total of 6 eligible studies (6517 cases and 16,886 controls) were included in the present meta-analysis. The results indicated that *MC4R* rs17782313 SNP was moderately associated with cancer risk (odds ratio = 1.12, 95% CI = 1.01–1.24). However, the subgroup analysis between different cancer types shows that rs17782313 is only associated with colorectal cancer but not the endometrial cancer and breast cancer. Risk factor in colorectal cancer was both significantly associated with rs17782313 with and without adjustment for body mass index; while the risk factor of the endometrial cancer and breast cancer were both not associated with the rs17782313 with and without adjustment for body mass index. There was no publication bias for the association between *MC4R* rs17782313 and cancer risk.

**Conclusion:**

The present meta-analysis confirmed the moderate association between *MC4R* rs17782313 and cancer risk.

## 1 Introduction

In 2008, the genome-wide association studies (GWAS) reported that rs17782313 single nucleotide polymorphism (SNP) mapped 188 kb downstream of the *melanocortin 4 receptor* (*MC4R*) gene was strongly associated with body mass index (BMI) and risk of obesity in European populations.^[1]^ Furthermore, subsequent studies have confirmed the positive association between SNPs in/near the *MC4R* gene and risk of obesity in populations with different races/ethnicities.^[2–4]^

MC4R is a 332-amino acid protein encoded by a single exon on chromosome 18q22. The rare coding mutations in the *MC4R* gene have been found to be the main cause of human monogenic obesity,^[5]^ suggesting that the *MC4R* gene represents a compelling biological candidate. MC4R expression is also associated with risk of early-onset obesity, increased lean mass and bone mineral density, and enhanced linear growth.^[6]^ Two previous meta-analyses confirmed that the rs17782313 SNP near the *MC4R* gene was associated with risk of obesity^[4]^ and type-2 diabetes.^[7]^ It has been well-documented that obesity is the leading risk factor for many cancers. Therefore, it is important to determine whether *MC4R* SNPs are associated with cancer risk, which may help illuminate the potential biological mechanism between obesity and cancer development. To date, several studies have investigated the associations of *MC4R* SNPs with risk of cancer.^[8–14]^ However, the findings have been contradictory.

The present study aimed to perform a systematic meta-analysis to clarify the association between the rs17782313 SNP (or its proxy) near the *MC4R* gene and risk of cancer.

## 2 Materials and Methods

### 2.1 Literature and search strategy

The PubMed and Embase databases were searched for potentially eligible studies. The following key words were used to search for eligible publications: (*melanocortin 4 receptor* OR *MC4R*) and (polymorphism OR variant OR variation OR genotype) and (cancer OR tumor OR carcinoma). The publication language was restricted to the English language. The reference lists of retrieved articles were also hand-searched. The literature search was updated as of September 10, 2019. Since this is a meta-analysis, ethical approval was waived.

### 2.2 Inclusion criteria and data extraction

The included studies met all the following inclusion criteria:

(1) studies that determined the association of *MC4R* rs17782313 (or its proxy SNP rs12970134, *r*^2^ > 0.90) with cancer risk;
(2) studies that had case-control design;
(3) studies that provided an odds ratio (OR) with 95% confidence interval (CI) with or without adjustments for BMI.

The following information were extracted from each study:

(1) name of the first author,
(2) year of publication,
(3) country of origin,
(4) race/ethnicity of the study population,
(5) number of cases and controls,
(6) gender ratio,
(7) mean age,
(8) mean BMI,
(9) cancer type,
(10) the studied SNP, and
(11) the determination of whether BMI was adjusted in the statistical model.

Two authors independently reviewed the articles for compliance with the inclusion/exclusion criteria, resolved any disagreement, and reached a consistent decision after discussion with a third author, if necessary.

### 2.3 Statistical analysis

The association between *MC4R* rs17782313 and cancer risk was determined by calculating the pooled OR and 95% CI under an additive genetic model. *Z*-test was used to determine the significance of the OR (*P* < .05 was considered statistically significant). Cochrane *Q*-test was conducted to assess the between-study heterogeneity.^[15,16]^*I*^2^ represented the range for the degree of heterogeneity. A random-effects model (DerSimonian–Laird^[15]^) was used to calculate the pooled OR when there was between-study heterogeneity (*P* ≤ .10 or *I*^2^ ≥ 50%). Otherwise, a fixed-effects model (Mantel–Haenszel^[16]^) was used. Publication bias was assessed by Begg test and Egger test^[17]^ (*P* < .05 was considered statistically significant). The data were analyzed using STATA version 11.0 (StataCorp LP, College Station, TX).

## 3 Results

### 3.1 Characteristics of the studies

**Figure 1:**
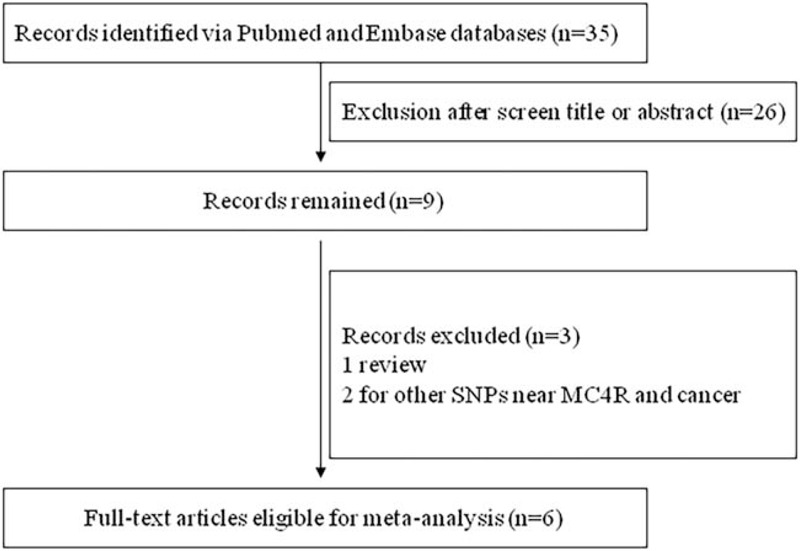
Flowchart for the inclusion/exclusion of studies.

Figure 1 presents a flow chart describing the process of inclusion/exclusion of studies. The literature search identified 35 potentially relevant articles. A total of 6 publications (6517 cancer cases and 16,886 healthy controls) were finally included in the present meta-analysis. The *MC4R* rs17782313 (or its proxy SNP rs12970134) in each included study was in Hardy-Weinberg Equivalent. The characteristics of the included studies are listed in Table 1.

**Table 1:**
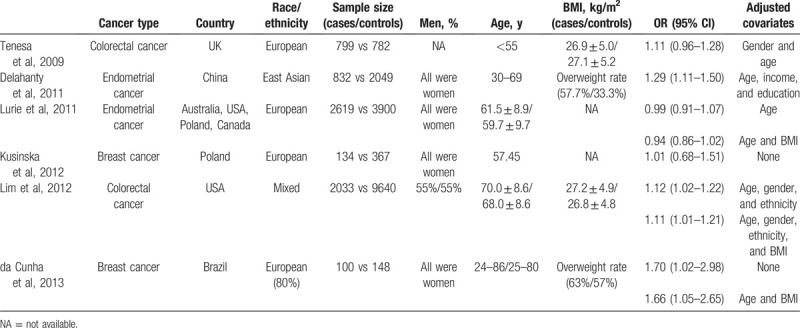
Characteristics of studies included in the meta-analysis.

### 3.2 Meta-analysis results

**Figure 2:**
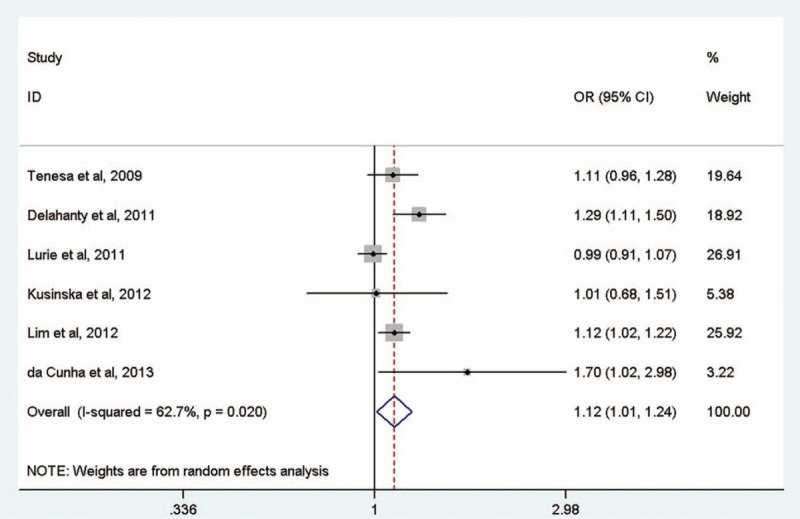
The meta-analysis of the association between *MC4R* rs17782313 and cancer risk without adjusting for body mass index.

Before adjusting for BMI, the *MC4R* rs17782313 SNP risk allele was moderately associated with cancer risk (OR = 1.12, 95% CI = 1.01–1.24) in an additive genetic model (Fig. 2). In the subgroup analysis by cancer type, there was a significant association with risk of colorectal cancer (OR = 1.12, 95% CI = 1.04–1.21). In contrast, the *MC4R* rs17782313 SNP was not associated with endometrial cancer (OR = 1.12, 95% CI = 0.87–1.45) or breast cancer (OR = 1.27, 95% CI = 0.77–2.11) (Table 2).

**Table 2:**
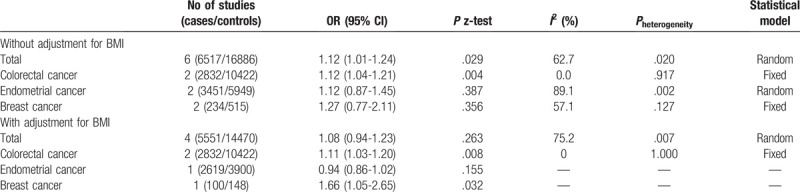
Meta-analysis of the association between *MC4R* rs17782313 and cancer risk by cancer type.

**Figure 3:**
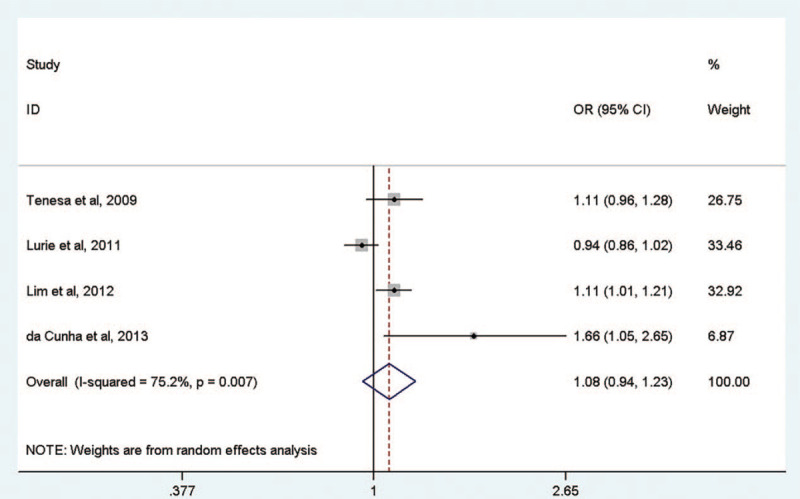
The meta-analysis of the association between *MC4R* rs17782313 and cancer risk with the adjustment for body mass index.

After adjusting for BMI, the *MC4R* rs17782313 SNP risk allele was not associated with cancer risk (OR = 1.08, 95% CI = 0.94–1.23; Fig. 3). In the subgroup analysis by cancer type, the *MC4R* rs17782313 SNP was moderately associated with the risk of colorectal cancer (OR = 1.11, 95% CI = 1.03–1.20; Table 2). While the risk factor of the other 2 cancer type (endometrial cancer and breast cancer) were both not associated with the rs17782313 with and without adjustment for BMI.

### 3.3 Publication bias

There was no publication bias for the *MC4R* rs17782313 SNP using Begg test (*P* = .452) or Egger test (*P* = .275) before adjusting for BMI, as well as after adjusting for BMI (*P* = .308 and.310, respectively).

## 4 Discussion

To our knowledge, this is the first meta-analysis that investigated the association between a SNP near the *MC4R* gene and risk of cancer. The present meta-analysis revealed that the *MC4R* rs17782313 SNP is moderately associated with risk of cancer, without adjusting for BMI. However, this association disappeared after adjusting for BMI. It appears that the association between the *MC4R* gene SNP and cancer risk may be mediated through adiposity.

Several previous GWAS have identified a large number of SNPs associated with obesity. The *FTO* gene is one of the first loci identified for obesity risk by GWAS. A most recent meta-analysis conducted by Kang et al revealed that the *FTO* gene rs9939609 SNP was not significantly associated with risk of cancer, regardless of the adjustment for BMI. However, in the subgroup analysis, this variant moderately increased the risk of endometrial cancer and pancreatic cancer, which was mediated by adiposity.^[18]^ Similarly, the authors also found a significant association between the *MC4R* gene rs17782313 SNP and risk of cancer, which mediated through BMI. Notably, a recent large-scale study suggested that *MC4R* gene SNPs are not associated with risk of colorectal cancer, regardless of adjusting for BMI.^[14]^ However, that study did not focus on the rs17782313 SNP, which is the interest of the present study.

The mechanism underlying the association between the *MC4R* SNP and cancer risk remains unclear. Similar to the *FTO* gene, the *MC4R* gene is also highly expressed in the central nervous system, which regulates the energy metabolism.^[19]^ It was reported that MC4R may regulate food choice and intake, and energy expenditure through a distinct pathway.^[20,21]^ However, further studies are needed to clarify the potential biological pathways through which these *MC4R* SNPs increase the risk of obesity and cancer.

It is important to focus on an organ system, which might encompass two or more different cancer types (eg, genitourinary cancer). However, merely 6 studies met the inclusion criteria. Thus, an analysis that focused on an organ system could not be performed. In addition, it is also important to assess the association between SNPs and different endocrine-driven cancers. However, due to the unavailability of data, subgroup analysis was performed by cancer type (colorectal cancer, endometrial cancer and breast cancer).

The present study had 2 strengths. First, the OR was extracted with 95% CI, with the adjustment of covariates from individual studies, to calculate the summary estimate, which represents an accurate estimate. Second, a total 6517 cancer cases and 16,886 healthy controls were included in the present meta-analysis, which greatly improved the statistical power. However, 2 limitations should be considered. First, although the total sample size was sufficiently large, merely 6 studies were included. In addition, the subgroup analysis by cancer type should be interpreted with caution due to the limited studies available for each cancer type. Second, there was a significant between-study heterogeneity in the meta-analysis, even although a random effects model was used to overcome this limitation. In addition, the further meta-regression analysis did not reveal any potential confounders that may explain the between-study heterogeneity.

In summary, there might be an association between the *MC4R* rs17782313 SNP and risk of cancer, which might be mediated by adiposity. Further studies are necessary to identify the causal variant near the *MC4R* gene, as well as the underlying mechanism between the *MC4R* gene SNP and risk of cancer.

## Author contributions

**Conceptualization:** Zeng Tian, Hongjun Xie.

**Data curation:** Xiaojiao Wang.

**Formal analysis:** Xiaojiao Wang.

**Investigation:** Zeng Tian.

**Methodology:** Jing Zhao, Yu Kang.

**Supervision:** Yu Kang.

**Validation:** Jing Zhao.

**Visualization:** Jing Zhao.

**Writing – original draft:** Zeng Tian, Jing Zhao, Hongjun Xie.

**Writing – review & editing:** Hongjun Xie.
